# IgG2 Antibodies against a Clinical Grade *Plasmodium falciparum* CSP Vaccine Antigen Associate with Protection against Transgenic Sporozoite Challenge in Mice

**DOI:** 10.1371/journal.pone.0111020

**Published:** 2014-10-24

**Authors:** Robert Schwenk, Margot DeBot, Michael Porter, Jennifer Nikki, Lisa Rein, Roberta Spaccapelo, Andrea Crisanti, Paul D. Wightman, Christian F. Ockenhouse, Sheetij Dutta

**Affiliations:** 1 Malaria Vaccine Branch, Walter Reed Army Institute of Research, Silver Spring, MD, United States of America; 2 3M Drug Delivery Systems, St. Paul, MN, United States of America; 3 Department of Experimental Medicine, University of Perugia, Perugia, Italy; 4 Imperial College London, London, United Kingdom; London School of Hygiene and Tropical Medicine, United Kingdom

## Abstract

The availability of a highly purified and well characterized circumsporozoite protein (CSP) is essential to improve upon the partial success of recombinant CSP-based malaria vaccine candidates. Soluble, near full-length, *Plasmodium falciparum* CSP vaccine antigen (CS/D) was produced in *E. coli* under bio-production conditions that comply with current Good Manufacturing Practices (cGMP). A mouse immunogenicity study was conducted using a stable oil-in-water emulsion (SE) of CS/D in combination with the Toll-Like Receptor 4 (TLR4) agonist Glucopyranosyl Lipid A (GLA/SE), or one of two TLR7/8 agonists: R848 (un-conjugated) or 3M-051 (covalently conjugated). Compared to Alum and SE, GLA/SE induced higher CS/D specific antibody response in Balb/c mice. Subclass analysis showed higher IgG2:IgG1 ratio of GLA/SE induced antibodies as compared to Alum and SE. TLR synergy was not observed when soluble R848 was mixed with GLA/SE. Antibody response of 3M051 formulations in Balb/c was similar to GLA/SE, except for the higher IgG2:IgG1 ratio and a trend towards higher T cell responses in 3M051 containing groups. However, no synergistic enhancement of antibody and T cell response was evident when 3M051 conjugate was mixed with GLA/SE. In C57Bl/6 mice, CS/D adjuvanted with 3M051/SE or GLA/SE induced higher CSP repeat specific titers compared to SE. While, 3M051 induced antibodies had high IgG2c:IgG1 ratio, GLA/SE promoted high levels of both IgG1 and IgG2c. GLA/SE also induced more potent T-cell responses compared to SE in two independent C57/BL6 vaccination studies, suggesting a balanced and productive T_H1_/T_H2_ response. GLA and 3M-051 similarly enhanced the protective efficacy of CS/D against challenge with a transgenic *P. berghei* parasite and most importantly, high levels of cytophilic IgG2 antibodies were associated with protection in this model. Our data indicated that the cGMP-grade, soluble CS/D antigen combined with the TLR4-containing adjuvant GLA/SE warrants further evaluation for protective responses in humans.

## Introduction

Malaria is a tropical disease that continues to cause wide spread mortality and morbidity in some of the poorest regions in the world. The development of a malaria vaccine could be a step towards eradication of this intractable disease. The major coat protein of *Plasmodium* sporozoites is the circumsporozoite protein (CSP). RTS,S (Glaxosmithkline, GSK) is a recombinant form of CSP that contains 18 NANP repeat units and the C-terminal cysteine rich region of *P. falciparum* CSP fused to the hepatitis B surface antigen and expressed in *Saccharomyces* yeast [1]. Early studies with an alum-adjuvanted RTS,S formulation showed only limited protection in humans. This could be due to the fact that alum induces primarily a T_H2_-type immune response [2], [3] and malaria infection is known to be at least partially controlled by cellular immunity [4]. Additionally, the magnitude and avidity of antibodies elicited by alum-based formulations may have been insufficient to induce protection. Protection was enhanced in humans when RTS,S was adjuvanted with the AS series of adjuvants that contained the T_H1_ inducers monophosphoryl lipid A or MPL (derived from bacterial cell wall lipopolysaccharide) and QS21 (a compound fractionated from the *Quillaja saponaria* tree). Vaccination with RTS,S+AS series adjuvants has since been shown to reproducibly protect ∼30–50% of vaccine recipients [5], [6] and protection has been associated with the induction of high levels of CSP-specific antibodies and cytokine producing (IL-2^+^ and IFN-γ^+^) CD4^+^ T cells [6], [7].

**Figure 1:**
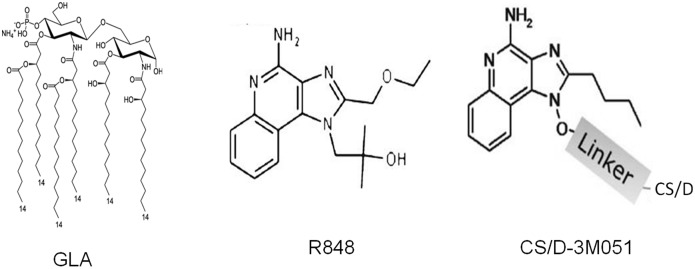
Molecular structure of TLR agonists GLA, R848, and CS/D-3M051 conjugate.

A potent adaptive immune response requires prior engagement of the innate immune system which can sense pathogen associated molecular patterns using pattern recognition receptors. The toll-like receptors (TLRs) are one of the best characterized families of pattern recognition receptors present on macrophages and dendritic cells [8]. In the mouse, TLRs 1, 2, 4, 5, 6 and 11 reside on the cell surface and interact with surface ligands while TLRs 3, 7, 8 and 9 are located in endosomes where they recognize pathogen nucleic acids [9]. Interaction of TLRs with their ligands activates a signal cascade terminating at transcription factors NF-kB and IRF-7 (MyD88 pathway) or IRF-3 (TRIF pathway) [10]. TLR signaling leads to the production of pro-inflammatory cytokines, type-1 interferons and chemokines which promote dendritic cell maturation and T_H1_ cell priming. TLR3 exclusively utilizes the TRIF pathway for downstream signaling while all other TLRs engage the MyD88 signal transduction cascade [11]. TLR4 is unique in its utilization of both the TRIF and MyD88 pathways [12]. Glucopyranosyl Lipid Adjuvant or GLA (Infectious Disease Research Institute, IDRI) is a potent TLR4 agonist (Fig. 1) and a homogenous synthetic analog of bacterial MPL [13]. Vaccines containing GLA have been tested clinically in nearly 1000 human subjects including those receiving pandemic flu vaccines [14], [15]. While MPL and GLA both promote robust T_H1_ responses, GLA has been reported to induce much stronger dendritic cell (DC) and peripheral blood mononuclear cell (PBMC) responses, and the addition of a stable oil-in-water emulsion (SE) to GLA (GLA/SE) further enhances these responses [16].

Single-stranded, viral RNA present within DCs binds to the TLR7 and TLR8 expressed on endosomal membranes [17]. Since native RNA degrades rapidly in serum a family of synthetic adjuvant compounds (imidazoquinolines) has been designed to mimic single-stranded RNA [18]. Imiquimod (R-837) has been used for topical immune therapy in millions of people in the commercial “Aldara” cream for treatment of external genital warts, basal cell carcinoma, and actinic keratosis. In addition, Orthoro *et al.* recently reported that the topical application of imiquimod at the site of subcutaneously injected *Plasmodium falciparum* circumsporozoite peptides elicited strong parasite-specific humoral immunity that protected against challenge with transgenic rodent parasites that express *P. falciparum* CSP repeats [19]. Structurally-similar TLR7/8 ligands such as resiquimod (R848), 3M-012 and 3M-051 (3M Drug Delivery Systems) are also under advanced consideration as vaccine adjuvants (Fig. 1) [20].

Elucidation of the molecular basis of antigen presentation is allowing for the development of more potent adjuvant formulations. Several groups are pursuing strategies that simultaneously target multiple TLRs. Such approaches take advantage of the differential location and signaling pathways used by various TLRs and their prevalence on different sub-populations of DCs [21]–[26]. Leishmaniasis immunotherapy studies show that a combination of MPL, a TRIF pathway-inducing TLR4 agonist, and CpG, a MyD88-associated TLR9 adjuvant, can synergistically increase the production of IL-12 and reduce the parasite burden in mice [27].

During an infection, TLR ligands and antigens are usually structurally linked and enter into the same antigen presenting cell [28]. Accordingly, another strategy of immune-enhancement is to chemically couple the TLR agonist to the antigen to allow simultaneous entry into the same DC. This approach leads to more efficient MHC class II presentation and T_H1_ differentiation [29]. Indeed, a covalent conjugate of HIV Gag protein with a TLR7/8 agonist, 3M-012, was found to be more immunogenic than a formulation containing unconjugated TLR7/8 agonist [30]. While 3M-012 utilizes UV light activated phenylazide chemistry for conjugation, its analog 3M-051 (used here) contains a reactive nicotinate hydrazide group that binds to aromatic aldehyde derivatized lysine residues on the antigen. Compared to 3M012, the 3M-051 coupling chemistry is more specific which makes it easier to quantify the coupling efficiency and adjuvant dose.

In an attempt to improve the protective efficacy of CSP-based vaccines, a nearly full-length recombinant CSP (CS/D) was produced in the *Escherichia coli* expression system under a GMP-compliant environment. We then tested this recombinant protein with adjuvant formulations containing the TLR agonists GLA, R848 and 3M-051 (Fig. 1). The magnitude of antibody response, subclass distribution, T cell response and protection against a PfCSP-transgenic *P. berghei* parasite challenge were monitored. The immunological outcome was highly dependent upon the adjuvant used, and the degree of protection correlated with the total IgG antibody titers as well as the level of induced cytophilic antibodies. These data form the basis for testing analogous formulations in humans.

## Materials and Methods

### Animals

Six- to eight-week old female Balb/c (H-2^d^) and C57Bl/6 (H-2^b^) mice were purchased from the Jackson Laboratories (Bar Harbor, ME). Animal procedures were conducted in compliance with the Animal Welfare Act and other federal statutes and regulations relating to animals and experiments involving animals and adhere to principles stated in the *Guide for the Care and Use of Laboratory Animals*, NRC Publication, 1996 edition. All procedures were reviewed and approved by the Walter Reed Army Institute of Research’s Animal Care and Use Committee (Protocol number:11-MVD-15), and performed in a facility accredited by the Association for Assessment and Accreditation of Laboratory Animal Care International.

### CSP Vaccine Antigen Production

**Figure 2:**
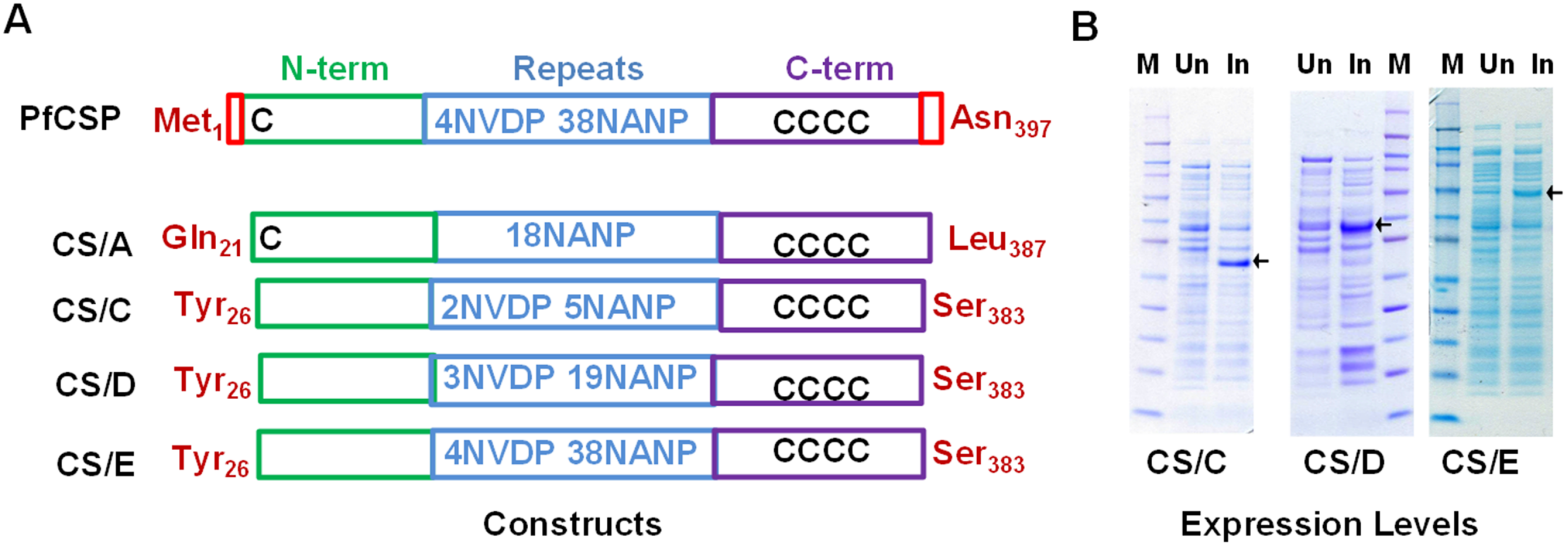
Recombinant CS/D constructs. *A,* Cartoon representation of the native *P. falciparum* CSP consisting of a signal sequence and GPI anchor sequence (red), N-terminal region (green), NVDP and NANP repeats (blue) and a cysteine rich C-terminal region (purple). The expressed constructs (CS/A, CS/C, CS/D and CS/E), their PfCSP-specific start and end residues, and the relative positions of the five cysteine residues (“C”) are shown**.** *B,* SDS-PAGE shows relative expression levels (arrows) in un-induced (Un) and induced (In) *E. coli* cells producing CS/C, CS/D and CS/E.

The 3D7-strain, *P. falciparum* CSP gene sequence (accession No. XP_001351122.1) was used to express several CSP constructs of varying repeat lengths (Fig. 2A). Based on the level of expression and the ability to protect mice against parasite challenge, a construct termed CS/D was chosen for process development. CS/D contained 19 NANP and 3 NVDP repeats and the majority of the N- and C-terminal regions of CSP (residues 26_Tyr_-127_Asp_ linked to 207_Pro_-383_Ser_). The gene used for expression was optimized by changing 180 of the 278 native codons to corresponding high abundance codons for *E. coli*. The AT content of the optimized gene was reduced from 65% to 50%. Expression cassettes were cloned in a pET32 plasmid (EMD Millipore, Billerica, MA) modified to allow kanamycin-based selection [31]. The CS/D-pET was transformed into the SHUFFLE (DE3) *E. coli* strain (NEB, Ipswich, MA) for protein expression.

Glycerol stocks of the CS/D-expressing clones were prepared in plant based, phytone-containing medium. To initiate GMP fermentation, a seed vial was inoculated into 3 L of medium and grown overnight at 32°C. This overnight culture was inoculated into 300 L of a medium containing BBL Phytone, (Sparks MD), yeast extract, ammonium sulfate, potassium and sodium phosphate, magnesium sulfate, glycerol, dextrose and kanamycin. The fermenter was set to 400 RPM agitation, 28°C, 300 L/min air flow, 3 psig pressure and pH 6.8. The culture was grown to an OD_600_ of 7.0 and induced with 0.5 mM IPTG for 2 h. The cell paste was harvested by centrifugation and stored at −80°C. For purification, the soluble fraction of the cell pellet was extracted in phosphate buffer by microfluidization, and the resulting lysate was clarified by centrifugation. CS/D protein was purified over a Ni-affinity column (Qiagen, Germantown, MD) and eluted using imidazole. The protein was then passed over a Q Sepharose fast flow ion-exchange column (GE Healthcare, Waukesha, WI) and the purified protein was eluted using a combination pH/salt step gradient. The final product was sterile filtered and stored at −80°C.

### Product characterization

Antigen purity was assessed by silver staining of SDS-PAGE gels (GelCode SilverSNAP Kit, Pierce). Host cell proteins were detected by ELISA using the Cygnus *E. coli* protein detection kit (Southport, NC) or by western blot using anti-*E. coli* antibodies from Dako (Carpinteria, CA). An analytical size-exclusion chromatography profile was obtained by loading 25 µg CS/D on a Shodex KW-803 column connected to a Waters HPLC system. N-terminal sequencing and mass spectroscopy was performed by Molecular Structure Facility, University of California (Davis CA). The endotoxin content of the final product was measured by the LAL Endotoxin Assay (Associates of Cape Cod, East Falmouth, MA). Sterility testing was performed by inoculating the protein bulk into culture media and evaluating for any bacterial growth. ELISA using CSP-specific monoclonal antibodies was used to establish product identity. In this monoclonal ELISA, the full-length CS/D protein, a repeat peptide: (NANP)_6_, a disulphide-bonded C term peptide: EPSDKHIKEYLNKIQNSLSTEWSPCSVTCGNGIQVRIKPGSANKPKDELDYANDIEKKICKMEKCS (Biomatik USA, Wilmington DE) and a reduced/alkylated version of this C-term peptide were coated on the ELISA plate.

### Rabbit Pyrogenicity Test

BioReliance (Rockville, MD) performed a rabbit pyrogen test to detect the presence of pyrogenic substances. Three adult rabbits (New Zealand white), weighing a minimum of 1.5 Kg received 3 mL/kg of diluted CS/D bulk (0.02 mL bulk +35 mL 0.9% saline) intravenously through the ear vein. The rectal temperature was measured at half-hour intervals from one to three hours. CS/D met the requirements for absence of pyrogens if no rabbit showed a temperature rise of 0.5°C or greater above its respective control temperature at any time period during the measurement.

### Antigens

A construct containing the C-terminal region of CSP (C-term) was cloned, expressed and purified to serve as a reagent in ELISA and Luminex assays. The Biotinlylated peptide NANP-(NANP)_5_-NANP-COOH (Luminex assays) and K^d^-restricted NYDNAGTNL peptide [32] (Balb/c cytokine assays) were synthesized by Alpha Diagnostics (San Antonio TX). Overlapping 15-mer peptides corresponding to the full-length CSP were a gift from Dr. Martha Sedegah (Infectious Disease Directorate, Naval Medical Research Center, Silver Spring MD). CSP-specific monoclonal antibodies (mAbs) were produced in a humanized mouse model and were selected based on their ability to react to sporozoite CSP by western blot and by IFA (Dr Ted B. Hall, *personal communication*).

### Adjuvants and Immunizations

Each vaccine dose contained 2.5 µg of purified CS/D. Conjugation of CS/D to the imidazoquinoline-derivative 3M-051 was performed by 3M Drug Delivery Systems (St. Paul, MN) using an NHS-Succinimidyl 4-formylbenzoate linker. The final conjugate vaccine was prepared using a 10 fold molar excess of 3M-051 to CS/D with 70% coupling efficiency. Each 2.5 µg dose of CS/D-3M051 contained 189 ng of 3M-051. Stable oil in water emulsion (SE), GLA/SE (0.25 mg/mL), GLA/R848/SE (0.25 mg/mL GLA+0.05 mg/mL R848), and Alum (1 mg/mL) were obtained from the Infectious Diseases Research Institute (IDRI, Seattle, WA). For all SE-based formulations, CS/D in phosphate-buffered saline (PBS; pH = 7.4) was mixed with the adjuvant (adjuvant: antigen v/v ratio 1∶4), the mixture was vortexed briefly, and 100 µl was injected subcutaneously (sc) into the inguinal region of each mouse. For Alum-based formulations, CS/D was mixed with Alum at a 1∶1 ratio (vol/vol), resulting in a formulation containing 50 µg Alum and 2.5 µg antigen injected sc into each mouse.

### Parasite Challenge and Protection

Protective efficacy was measured using a transgenic (Tr-Pb) spz challenge model [33], [34]. The transgenic *P. berghei* (Pb) parasite expresses the full-length *P. falciparum* CSP [35]. Our initial immunogenicity studies were carried out in Balb/c mice. However, it became apparent that C57Bl/6 mice are much more susceptible than Balb/c mice to infection with the Tr-Pb spz, and hence all challenge experiments were conducted using the C57Bl/6 strain. C57Bl/6 mice were given either two or three vaccinations and challenged intravenously (iv) with approximately 3000 spz two weeks after the final immunization, as described by Porter *et al.* [33]. Protection was defined as the absence of blood stage infection 14 days post-challenge as observed via oil immersion microscopy of a Giemsa stained thin smear.

### Reagents

CSP specific monoclonal antibodies used to characterize recombinant CSP were generated in mice against a full-length CSP recombinant protein [36] and showed positive reactivity to Pf sporozoites by IFA and western blot (Dr. Ted B. Hall, personal communication). PE-anti-mouse IgG, PE-anti-mouse IgG1, PE-anti-mouse IgG2c and PE-anti-mouse IgG2a were obtained from Jackson Immunoresearch (West Grove, PA). Luminex beads and Luminex Streptavidin beads were purchased from Luminex Corporation (Austin, TX) and 96-well Luminex Multi-screen BV 1.2 micron assay plates were acquired from Millipore Corporation (Billerica, MA). Antigens were coupled to the Luminex beads according to the manufacturer’s instructions. PerCP anti-CD3 (clone 145-2C11), Pacific Blue anti-CD4 (clone RM4-5), Horizon V500 anti-CD8 (clone 53-6.7), Alexa 700 anti-CD44 (clone IM7), FITC anti-IL-2 (clone JES6-5H4), APC anti-IFN-γ (clone XMG1.2), anti-CD16/CD32 FcR Block (clone 2.4G2), anti-CD28 (clone 37.51), anti-CD49d [clone 9C10(MFR4.B)], Cytofix/Cytoperm and Golgi Plug were all obtained from BD Biosciences (San Jose, CA). LIVE/DEAD Fixable Blue Dead Cell Stain Kit for UV excitation was purchased from Invitrogen (Camarillo, CA). Phorbol 12-myristate 13 acetate (PMA) and Ionomycin were obtained from Sigma Chemicals (St. Louis, MO).

### ELISA

Immulon 2HB plates (Thermo Scientific, Rochester, NY) were coated overnight at 4°C with either 50 ng/well recombinant CS/D or 20 ng/well (NANP)_6_ peptide. Plates were washed with PBS containing 0.05% Tween-20 (PBS/T) and blocked with PBS containing 1% casein for 1 h. 100 µl of serially diluted primary antibody was added to the wells in duplicate for 2 h at 22°C, the plates were washed 3x with PBS/T, and 50 µl of a 1∶15,000 dilution of HRP-conjugated, anti-mouse IgG (Southern Biotech, Birmingham, AL) was added per well. After a 1 h incubation, the plates were washed 4x with PBS/T and developed using 50 µl/well ABTS peroxidase substrate system (KPL, Gaithersburg, MD) for 1 h. OD_415_ was measured using a Biotek Synergy 4 microplate reader (Highland Park, VT), and endpoint titer, defined as the serum dilution that resulted in an OD_415_ of 1.0, was calculated using Gen5 software (Biotek). For the monoclonal ELISA, serial dilution of humanized-mouse monoclonal antibodies against CSP (starting at 1∶500 dilution of 1 mg/ml mAb) were added to the wells and the ELISA was developed as described above using human secondary antibody.

### Avidity ELISA

The avidity ELISA protocol was similar to that described above, except an additional wash step was added involving incubation with either 6 M Urea or PBS for 10 min following the primary antibody binding step. Avidity index was defined as the percentage of antibody that remained bound to antigen after the urea wash = (endpoint titer with urea wash)/(endpoint titer with PBS wash)×100.

### Luminex

To determine serum dilutions for the Luminex analysis (Luminex Corporation, Austin, TX), serum from several representative high and low responder mice were first analyzed. These sera were serially diluted and MFI response measured by Luminex. Titration curves were drawn and a dilution that fell on the linear part of the curve for most samples was chosen. A representative titration experiment is shown in Figure S1. Subsequently, all serum samples were run at this one selected dilution. Mouse serum was collected at 1 month (Balb/c) or 2 weeks (C57Bl/6) after the final immunization and diluted to 1/500 (Balb/c) or 1/2000 (C57Bl/6) in PBS containing 1% BSA (assay buffer). 50 µl of the serum plus assay buffer mixture was added to the wells of a Luminex plate followed by 50 µl of assay buffer containing 3000 CS/D-coupled beads or 3000 beads coupled with C-term protein plus 3000 beads (with a different bead signature) coupled to NANP. The plates were agitated on a shaker for 1 h at room temperature and washed in assay buffer containing 0.05% Tween-20. 100 µl of assay buffer containing PE-labeled-(mouse IgG subclass)-specific antibody was then added to the wells and the plate was agitated for an additional hour. The plate was washed and mean fluorescence intensity (MFI) was measured using a Luminex 200 system (Luminex Corporation, Austin, TX).

### Cytokine Production Assay

To allow for the development of stable memory cells, spleens were obtained from either Balb/C or C57Bl/6 mice at least six weeks after the final immunization. Spleen cells (10^6^ per well) were cultured with anti-CD28 (1 µg/ml) and anti-CD49d (1 µg/ml) co-stimulants plus either medium (negative control), CS/D protein (10 µg/ml), K^d^-restricted CD8 T cell peptide (10 µg/ml) or a pool of overlapping 15-mer peptides covering the entire CSP sequence (2 µg/ml). Naïve T cells cultured with CS/D served as an additional negative control, and PMA/Ionomycin was used as the positive control. Cells were incubated for 2 h at 37°C in a 96-well round-bottom culture plate and Golgi plug was added for an additional 4 h at 37°C. Cells were stored at 4°C overnight and then transferred to a 96-well, V-bottom plate for surface-staining of CD3, CD4, CD8, and CD44. Subsequently, cells were washed and fixed/permeabilized simultaneously with Cytofix/Cytoperm. Cells were stained for intra-cellular IFN-γ and IL-2, washed, and a minimum of 300,000 cells per sample were acquired on an LSR II multicolor flow cytometer (BD Biosciences). A typical gating scheme for the detection of cytokine producing CD4^+^ and CD8^+^ T cells is shown in Figure S2. Viable lymphocytes were gated for CD3^+^ cells and further gated to reveal CD4^+^ and CD8^+^ T cells. The CD4^+^ population was differentiated by the presence of CD44 to reveal activated CD4^+^ T cells producing cytokines. Data was analyzed using FlowJo software (Tree Star, Ashland, OR).

### CD86 expression assay

Splenic lymphocytes from naïve mice were incubated overnight with medium alone (negative control), 1 µg/ml of CS/D, 1 µg/ml CS/D-3M051, or 1 µg/ml TLR ligand-containing adjuvants. The cells were washed and stained for surface expression of CD3, CD19, and CD86 and flow cytometry data was acquired as above. Viable lymphocytes were gated to reveal CD19^+^ B Cells. Data was analyzed using FlowJo software.

### Statistics

ELISA and Luminex data were log transformed and two-way comparisons were made by unpaired *t*-test on the GraphPad Prism software (La Jolla, CA). Multiple comparisons were made by ANOVA and p-values were corrected using Tukey’s method. Statistically significant difference in group means was indicated in figures as **** (p<0.0001), *** (p<0.001), ** (p<0.01), or * (p<0.05). Parasite challenge data on day 14 were analyzed by the Fisher’ exact test. Survival curves were compared using log rank test and Dunnett's method was used to adjust p values for multiple comparisons (SAS software version 9.3).

## Results

### Expression and characterization of CS/D in *Escherichia coli*

*P. falciparum* 3D7 strain CSP is composed of the N-terminal region which harbors one cysteine residue, followed by 4 NVDP and 38 NANP repeats and a C terminal region that harbors four additional cysteines that form two disulphide bonds (Fig. 2A) [37]. At the time of hepatocyte invasion, the N-terminal region of CSP is proteolytically processed [38], but the exact processing site has not been mapped. Our expression constructs were designed to include a majority of the N-terminal region of PfCSP. The construct CS/A (Fig. 2A) that contained all 5 cysteines, resulted in a protein that aggregated at low concentrations and was not stable during freeze-thaw cycles. All subsequent constructs were therefore designed to exclude the first cysteine, initiating expression at Tyr_26_ (Fig. 2A). In order to optimize the number of repeats in the antigen, we produced three proteins: CS/C (2NVDP+5NANP repeats), CS/D (3NVDP+19NANP) and CS/E (4NVDP+38 NANP) (Fig. 2A). CS/E contained all PfCSP repeats but it expressed at ∼3% of total cell protein, in contrast, CS/C and CS/D (which contained fewer repeats) expressed at ∼14% of total cell protein (Fig. 2B). In a mouse immunogenicity study, the CS/C protein produced very low level NANP-specific antibodies and failed to induce protection against transgenic *P. berghei* challenge [33] and hence the process development efforts were focused on the CS/D construct. CS/D protein partially partitioned into the soluble fraction of *E. coli* and the soluble protein was extracted and purified using a 2-step process.

### Product characterization

**Figure 3:**
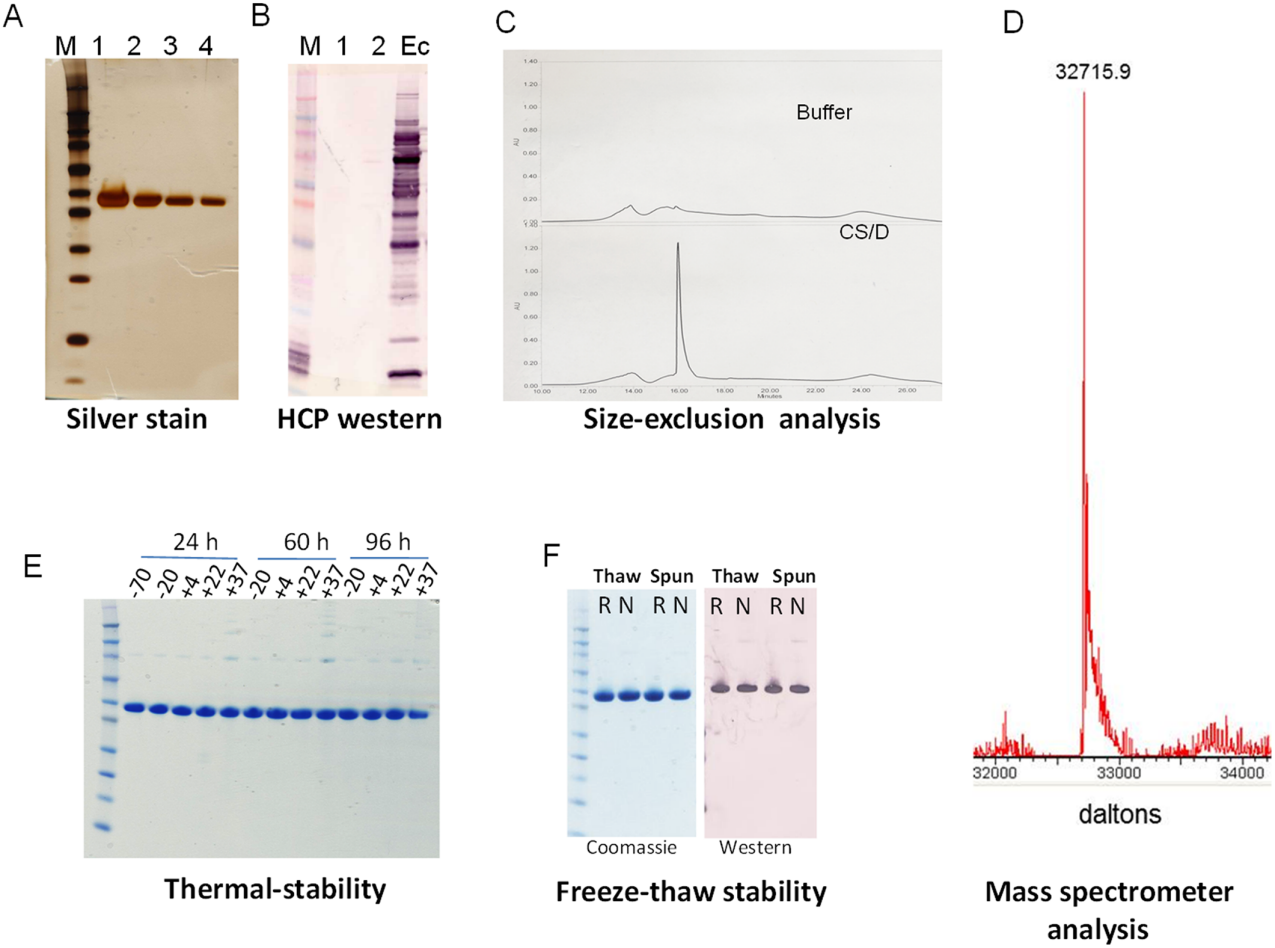
Characterization of CS/D product. *A,* Purity of CS/D final product analyzed by silver-stained SDS-PAGE. Lanes, 1, 2, 3 and 4 show 2 µg, 1 µg, 0.5 µg, 0.25 µg of CS/D, respectively; M, molecular weight marker. *B,* Western blot using host cell protein polyclonal antibodies. Lane 1 shows 2 µg CS/D, lane 2 shows 1 µg CS/D; Ec, whole cell *E. coli* lysate positive control; M, molecular weight marker. *C,* Size-exclusion HPLC elution profile of CS/D. *D*, Mass spectrometry analysis of CS/D. *E*, Thermal Stability of CS/D shown on a non-reduced SDS-PAGE with temperature and incubation time shown above the gel. *F*, Stability of CS/D following a freeze-thaw cycle. Frozen and thawed samples (Thaw) were centrifuged (Spun) were analyzed by coomassie-stained, reduced (R) and non-reduced (N) SDS-PAGE and by mouse polyclonal anti-CS/D western blot.

A summary of the results of various tests performed on the final product is shown in Table 1. The final product contained >99% pure CS/D protein, as determined by silver-staining of SDS-PAGE gels (Fig. 3A). No *E. coli*-specific bands were detected by western blot using host cell protein antibodies (Fig. 3B), and using a more sensitive host cell-protein ELISA revealed that 400 µg/ml bulk protein contained 4 ng/ml bacterial proteins. On an analytical size-exclusion column, CS/D eluted as a single peak, confirming the conformational homogeneity of the product (Fig. 3C). N-terminal sequence of CS/D protein was AHHHHHHPGM**YGSSSNT**, confirming the processing of the terminal methionine, the presence of a 6xHis tag and 7 CSP specific residues (Table 1, in bold). Mass spectrometry analysis showed one peak at 32,715.9 Da which is close to the predicted mass of 32,717.9 Da (Fig. 3D). The endotoxin content of CS/D was 6 EU/ml (0.015 EU/µg protein) as measured by the Pyrochrome LAL kit and the product was non-pyrogenic in rabbits. The final product was sterile filtered and it passed the sterility test. The protein was stable at temperatures below 22°C for up to 96 h. Aggregation was noticeable after 60 h incubation at 37°C and some degradation was observed after 96 h at 37°C (Fig. 3E). No loss of product due to precipitation was observed when CS/D samples were frozen, thawed and analyzed by SDS-PAGE/western blot, before or after centrifugation (Fig. 3F).

**Table 1:** Specifications of cGMP-grade CS/D product.

Characteristic	Result	Method
Protein concentration	0.4 mg/ml	BCA method
Purity	>99%	Densitometry of silver stained gel
Host cell protein content	4 ng/ml	Cygnus host cell ELISA
Host cell protein detection	Negative	DAKO anti-*E. coli* western blot
Molecular weight	∼35 kDa	SDS-PAGE
Molecular weight	32,716	Mass spectrometry
Identity	Positive	CSP mAb ELISA/Western blot
N-terminal sequence	AHHHHHHPGMYGSSSNT	Edman sequencing
Sterility	Sterile	Inoculation method
Stability	Stable for 60 hr at 22°C	SDS-PAGE
Endotoxin	<6 EU/ml (0.001 EU/mg)	LAL Pyrochrome Assay
Pyrogenicity	Non-pyrogenic	Rabbit pyrogenicity test

**Figure 4:**
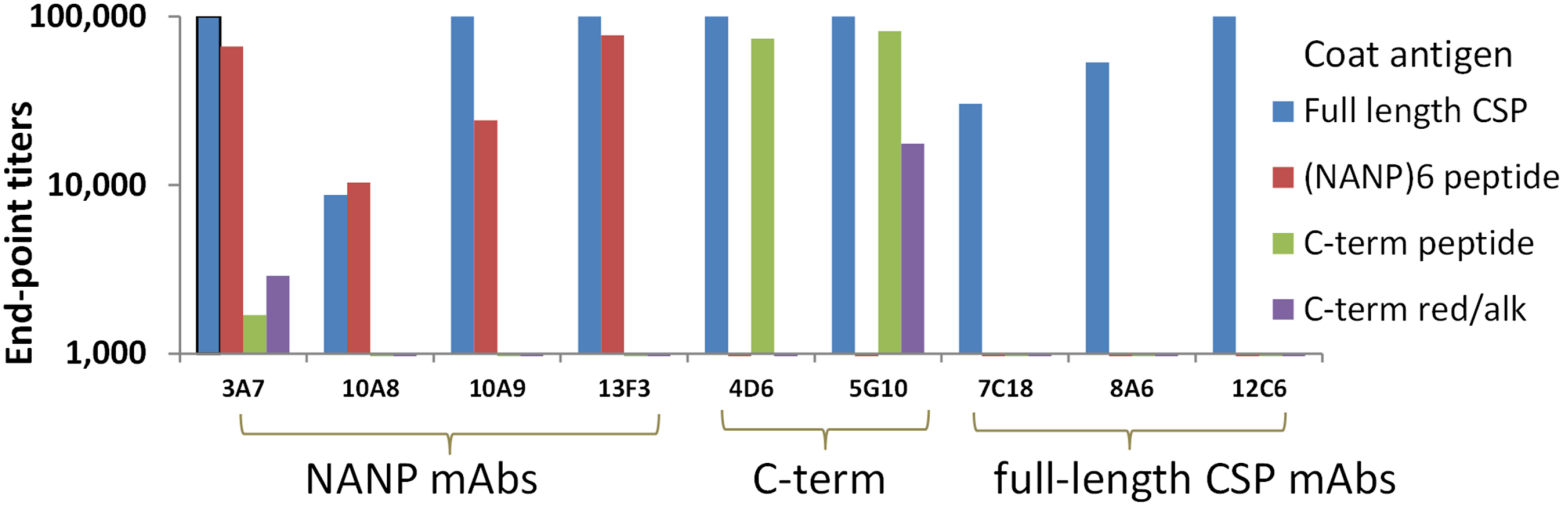
ELISA end-point titers of CSP mAbs. Full-length CS/D protein, repeat (NANP)_6_ peptide and non-reduced or reduced/alkylated (red/alk) version of a C-term peptide (EPSDKHIKEYLNKIQNSLSTEWSPCSVTCGNGIQVRIKPGSANKPKDELDYANDIEKKICKMEKCS) was coated in wells and mAb dilution that resulted in an OD = 1.0 was plotted.

The immunological identity of CS/D was established by ELISA against a panel of CSP-specific monoclonal antibodies (mAbs) (Fig. 4). MAbs 3A7, 10A8, 10A9 and 13F3 bound to the NANP repeat region. Mabs 4D6 and 5G10 bound to a conformation-dependent epitope represented by a disulphide-bonded C-term peptide. Significantly lower mAb reactivity was observed when this C-term peptide was reduced/alkylated to break the disulphide bonds using DTT/iodoacetamide. Three mAbs 7C18, 8A6 and 12C6 recognized epitopes present only within the full-length CS/D. The final CS/D product showed high level reactivity to all of the above CSP-specific mAbs (Fig. 4).

### Effects of TLR agonists on immunogenicity of CS/D in Balb/c mice

**Figure 5:**
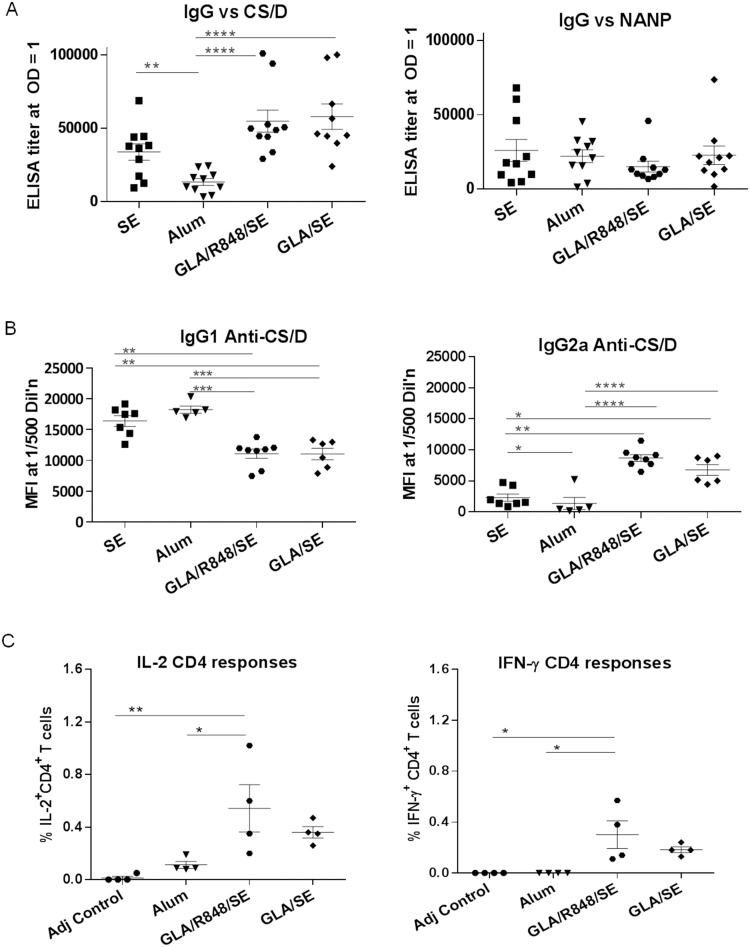
Immunological responses induced in Balb/c mice by vaccine formulations using SE, Alum or TLR ligands. Groups of 10 Balb/c mice were immunized with 2 doses of 2.5 µg CSP, 2 months apart in the indicated adjuvant. Two weeks post second immunization, the mice were bled and the serum was analyzed. *A,* ELISA end point titers of Balb/c mice, measured against CS/D (left) or NANP repeat peptide (right). *B,* IgG1 (left) and IgG2a (right) subclasses measured by Luminex and expressed as mean fluorescence intensities (MFI) at 1∶500 serum dilution. *C,* Percentage of total cytokine^+^CD44^+^CD4^+^ T lymphocytes, extracted from vaccinated Balb/c mice and stimulated with CS/D, stained for surface expression of CD3, CD4 and CD44 and intra-cellular expression of IL-2 (left) or IFN-γ (right). Lines are mean with SEM. (*) indicates significant P values for ANOVA followed by Tukey’s multiple comparison test.

We have previously reported that CS/D adjuvanted with Montanide ISA720 at a 2.5 µg dose gave low level protection (20–30%) in the transgenic parasite challenge model [33]. Hence, immunizations in the current study were conducted at the sub-saturating, 2.5 µg dose of GMP CS/D. In the first experiment, a group of 10 Balb/c mice received two immunizations of CS/D, at two month intervals, using four different adjuvants: SE, Alum, GLA/R848/SE, GLA/SE. The adjuvant control group received PBS+GLA/R848/SE. The full-length, CS/D-specific ELISA titers of the GLA/SE and GLA/R848/SE groups did not differ but both were higher than the Alum group (Fig. 5A). The central NANP repeat titers were not different between vaccine groups. Subclass analysis revealed that the TLR agonist-containing groups GLA/SE and GLA/R848/SE induced 3-fold higher IgG2a and lower 1gG1 than SE (Fig. 5B). To assess T cell responses, CS/D-specific IL-2^+^ and IFN-γ^ +^ cells were measured as a percentage of total CS/D-specific CD44^+^ CD4^+^ T cells. GLA/R848/SE induced higher T cell responses than the adjuvant control and the Alum group (Fig. 5C). No IL-2 or IFN-γ-producing CD8^+^ T cells were detected in this immunization study.

The CS/D ELISA titers of the alum group were lower than those induced by SE (Fig. 5A) and the subclass pattern and T cell responses induced by Alum were similar to SE (Fig. 5B, C). Therefore, alum formulations of CS/D were not included in any subsequent immunogenicity studies. Overall, the addition of the TLR agonist GLA to the SE formulation was found to augment CS/D-specific antibody production, IgG2a levels and a trend towards enhanced CS/D-specific T-cell responses. Vaccination with the GLA/R848/SE formulation resulted in a larger spread in the levels of cytokine-producing CD4^+^ T cells as compared to GLA/SE, but no overall improvement in immunogenicity was observed when the unconjugated TLR7/8 ligand R848 was added to GLA/SE (Fig. 5).

### Immunogenicity of CS/D covalently conjugated to a TLR7/8 agonist

**Figure 6:**
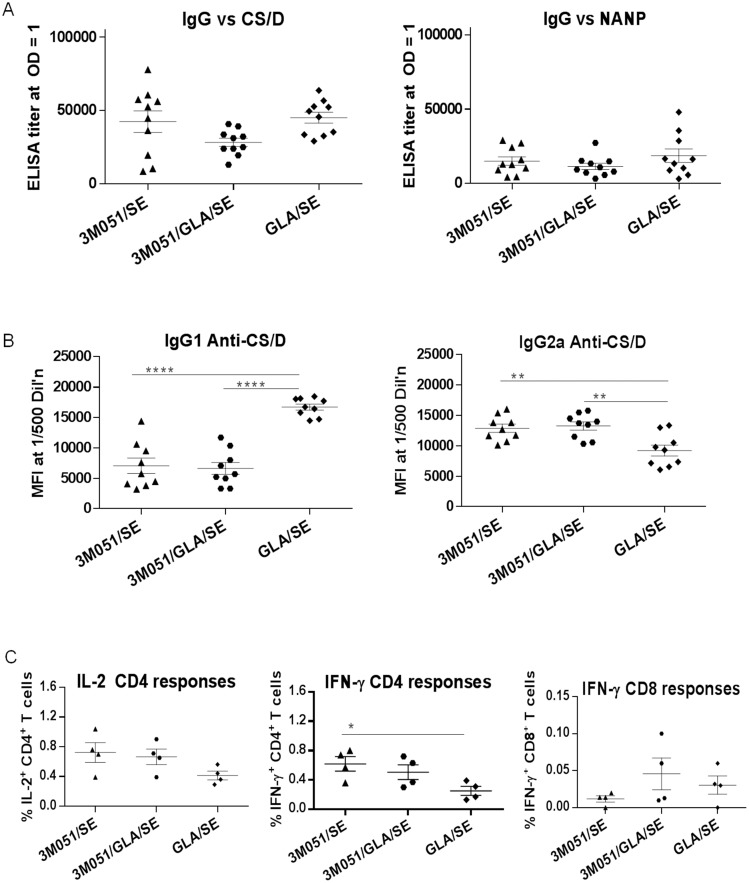
Immunological responses induced in Balb/c mice by CS/D covalently conjugated to 3M-051. Groups on 10 Balb/c mice were immunized two doses of 2.5 µg CSP, 2 months apart in the indicated adjuvant. One month post second immunization sera were collected and analyzed. *A,* ELISA end point titers of mice, measured against CS/D (left) or NANP repeat peptide (right). *B,* IgG1 (left) and IgG2a (right) subclasses expressed as Luminex mean fluorescence intensities (MFI) at 1∶500 serum dilution. *C*, Percentage of total Cytokine^+^CD44^+^CD4^+^ T lymphocytes, extracted from vaccinated Balb/c mice and stimulated with CS/D, stained for intracellular IL-2 (left) or IFN-γ (middle) or CD8^+^ cells stimulated with the K^d^-restricted peptide and stained for IFN-γ (right). Lines are mean with SEM.

In order to determine whether the adjuvant activity of TLR7/8 agonists would improve as a result of co-internalization with CS/D, we tested the immunogenicity of a covalently conjugated TLR7/8 ligand 3M051. The 3M051 conjugate was formulated with either SE or GLA/SE and its immunogenicity was compared to CS/D formulated in GLA/SE. The CS/D- and NANP-specific IgG antibody responses of the two 3M051 groups were similar to the GLA/SE (Fig. 6A). At the subclass level, 3M051, either in SE or combined with GLA/SE, induced higher IgG2a and lower IgG1 than GLA/SE (Fig. 6B). Although the T cell analysis was limited by low numbers of mice in each group there was also a trend towards higher IFN-γ^ +^ CD4^+^ T cell responses in the 3M051 groups as compared to GLA/SE (Fig. 6C). Additionally, a weak IFN-γ^+^ CD8^+^ T cell response was noted in all vaccine groups (Fig. 6C). Conjugation of CS/D to 3M051 promoted a greater T_H1_ bias than GLA/SE. However, the overall immunogenicity of the 3M051 conjugate was similar to GLA/SE and no improvement was observed when the two were combined, and hence mixtures of TLR agonists were not evaluated further.

### Effect of TLR agonists on CS/D immunogenicity in C57Bl/6 mice

**Figure 7:**
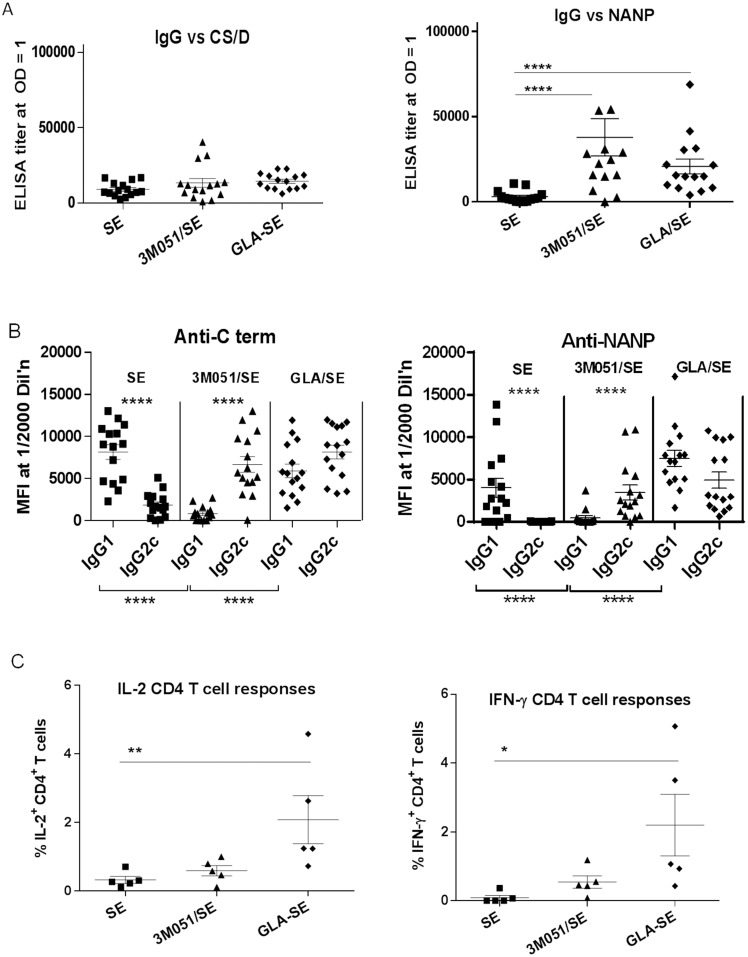
Immunological responses induced in C57Bl/6 following 2 vaccinations with CS/D adjuvanted with GLA/SE or 3M051. Groups on 15 C57Bl/6 mice were immunized with 2 doses of 2.5 µg of CSP, 3 weeks apart, in the indicated adjuvant. Two weeks post second immunization sera were analyzed. *A,* ELISA end point titers of mice, measured against CS/D (left) or NANP repeat peptide (right). Two high responding mice from the 3M051/SE conjugate group were included in statistical analysis but are outside the scale of the NANP graph. *B,* Relative IgG1 *vs.* IgG2c responses against the C term protein (left) or NANP peptide (right) measured by Luminex at 1∶2000 serum dilution. *C,* Percentage of total Cytokine^+^CD44^+^CD4^+^ T lymphocytes, extracted from vaccinated C57Bl/6 mice and stimulated with CS/D, stained for intracellular IL-2 (left) or IFN-γ (right). Lines are mean with SEM.

The 3M051-induced shift towards IgG2 and a trend towards higher IFN-γ T cell responses in Balb/c mice prompted us to compare the efficacy of this 3M051 conjugate to the efficacy of CS/D formulated in GLA/SE against a transgenic parasite challenge. As described in the methods section, Balb/c mice were somewhat resistant to infection with transgenic parasites, and hence the challenge experiments were carried out in the more susceptible C57Bl/6 strain. This set of experiments also allowed us to investigate the immune responses in an alternative genetic background with different T_H1_/T_H2_ predispositions. Two immunizations with 2.5 µg CS/D formulated with SE or GLA/SE or conjugated to 3M051/SE were given to groups of 15 mice at a one month interval. Two weeks after the second immunization, the ELISA titers against CS/D were similar between groups, but the NANP repeat titers induced by the TLR-containing formulations 3M051/SE and GLA/SE were significantly higher than those of SE (Fig. 7A). When IgG subclass analysis was dissected into NANP and C-term responses, each of the three adjuvants showed a unique IgG2 *vs.* IgG1 profile. 3M051/SE induced high IgG2c levels and almost no IgG1, whereas the SE formulation showed the opposite subclass distribution (low IgG2 and high IgG1) to both C term and NANP. In contrast, GLA/SE induced similar levels of IgG2 and IgG1 antibodies (Fig. 7B). Overall, the 3M051 conjugate induced significantly lower levels of IgG1 as compared to SE or GLA/SE-adjuvanted CS/D. Spleens from 5 of the 15 immunized mice were harvested for T cell analysis. The IL-2 and IFN-γ CD44^+^ CD4^+^ T cell responses of the GLA/SE group were higher than SE, while T cell responses in the 3M051/SE group were not statistically different from SE (Fig. 7C).

**Figure 8:**
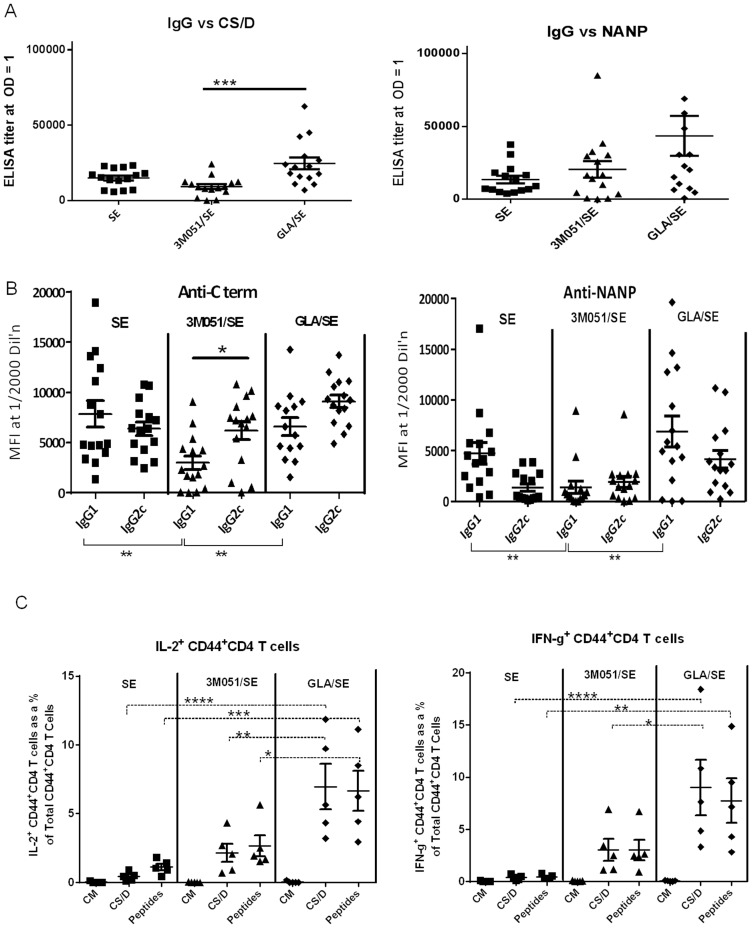
Immunological responses induced in C57Bl/6 following 3 vaccinations with CS/D adjuvanted with GLA/SE or 3M051. Groups of 15 C57Bl/6 mice were immunized three times, 3 weeks apart, with 2.5 µg of CSP in the indicated adjuvant. Sera were analyzed 2 weeks after the last dose. *A,* ELISA titers of mice measured against CS/D (left) and NANP peptide (right). Two high responding mice from the GLA/SE group were included in statistical analysis are outside the scale of the NANP graph. *B,* Relative IgG1 *vs.* IgG2c responses against the C term protein (left) or NANP peptide (right) measured by Luminex at 1∶2000 serum dilution. C, Percentage of total Cytokine^+^CD44^+^CD4^+^ T lymphocytes, extracted from vaccinated C57Bl/6 mice and stimulated with CS/D, stained for intracellular IL-2 (left) or IFN-γ (right). Lines are mean with SEM.

In another study three immunizations with 2.5 µg CS/D formulated with SE or GLA/SE or conjugated to 3M051/SE were given to groups of 15 mice at 3 week interval. Unlike the two dose study, higher ELISA titers against CS/D were induced by GLA/SE as compared to 3M051/SE (Fig. 8A). The 3M051 conjugate again induced lower levels of IgG1 than SE or GLA/SE-adjuvanted CS/D, but the unique IgG2 *vs.* IgG1 profile of 3M051/SE observed in the two dose study (high IgG2c and almost no IgG1) was evident only for the C-term responses (Fig. 8B). A balanced T_H1_/T_H2_ response characterized by similarly high levels of IgG2 and IgG1 antibodies against C-term and NANP was once again confirmed for the GLA/SE group (Fig. 8B). T cell analysis performed on 5 of the 15 immunized mice also confirmed that GLA/SE was a more potent adjuvant than SE and 3M051/SE (Fig. 8C). Antibody avidity against CS/D- or NANP was measured but it showed no difference between groups (data not shown).

### Effect of TLR agonists on protective efficacy against Tr-Pb sporozoite challenge

**Figure 9:**
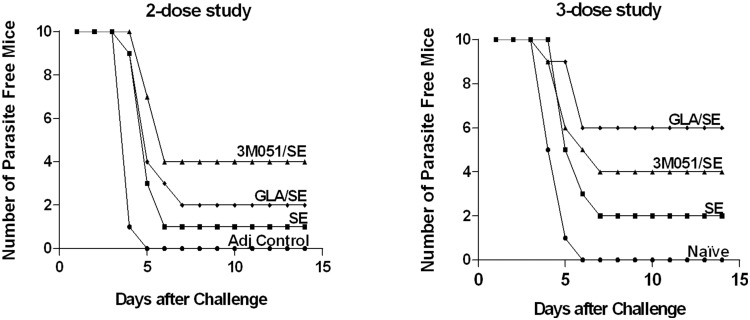
Protective efficacy in C57Bl/6 mice against transgenic *P. berghei* sporozoite challenge. *A,* Survival curves of C56Bl/6 mice (n = 10), challenged with the transgenic parasites in the 2-dose (left) or 3-dose (right) study. Protection was defined as the absence of blood stage infection until day 14 post challenge.

The remaining 10 C57Bl/6 mice from the two immunization experiments were challenged intravenously with 3000 transgenic sporozoites two weeks after the final immunization. In the two dose study, all the mice in the adjuvant control group (PBS+SE) developed patent blood-stage malaria by five days post-infection. In contrast, 4/10 mice in the 3M051/SE group, 2/10 mice in the GLA/SE group and 1/10 mice in the SE group remained sterilely protected at 14 days post-challenge (Table 2). The log rank test was used to compare survival time of mice between experimental groups and Dunnett’s method was used to adjust P values for multiple comparisons. There was a significant difference in survival time between experimental groups for the 2 dose study (Chi-square = 28.11, DF = 3, P<0.0001). More specifically, the survival time for the control group was less than CSP adjuvanted with GLA (adjusted P = 0.0060), 3M051/SE (P<0.0001), and SE (P = 0.0360) (Fig. 9).

**Table 2:** C57Bl/6 mouse protection data following two or three immunizations with CS/D combined with multiple adjuvants.

Adjuvant	CS/D	Protection	
	µg	2-dose	3-dose
3M051	2.5	4/10	4/10
GLA/SE	2.5	2/10	6/10
SE	2.5	1/10	2/10
SE	0	0/10	N/A
Naïve	0	N/A	0/10
GLA/SE	0.1		1/7
GLA/SE	1		4/7
GLA/SE	2.5		6/7
GLA/SE	5		5/7
GLA/SE	10		6/7
GLA/SE	0		0/7

In the three-dose study, all naïve controls developed blood-stage malaria by day six post-infection but 4/10 mice in the 3M051/SE group, 6/10 mice in the GLA/SE group and 2/10 mice in the SE group remained sterilely protected on day 14 post challenge (Table 2). As in the first challenge study a significant difference in survival time between experimental groups was observed (Chi-square = 18.11, DF = 3, P = 0.0004) and survival time for the control group was less for CSP adjuvanted with GLA (P = 0.0004) and 3M051 (P = 0.0130) (Fig. 9). In both studies, the protective efficacy at the end of the trial for CSP adjuvanted with GLA/SE and 3M051/SE were not statistically significant (Fischer’s exact test). The difference in protection, 2/10 and 6/10 in the GLA/SE group of the 2-dose and 3-dose studies respectively was reflected in the higher CS/D titers (∼15000 *vs.* ∼25000) (Fig. 7A and 8A).

**Figure 10:**
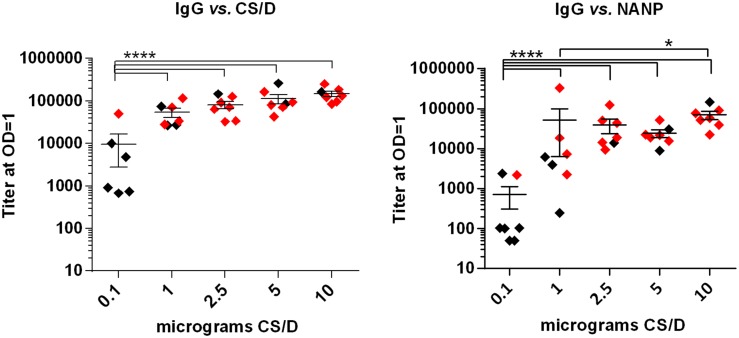
Dose response study of CS/D+GLA/SE vaccine in C57Bl/6 mice. Groups of 7 mice received three immunizations of 0.1, 1, 2.5, 5 or 10 µg CS/D, adjuvanted with GLA/SE at two week intervals. ELISA end-point titers were measured 2 weeks after the last dose against CS/D (left) or NANP (right). Red circles represent protected mice while black circles represent non-protected mice.

Based on the above data, the GLA/SE formulation was selected for further evaluation in the challenge model. Three doses of 0.1, 1, 2.5, 5, or 10 µg of CS/D formulated in GLA/SE were administered to 7 C57Bl/6 mice at 2 week interval. Two weeks after the third immunization, ELISA titers generally increased with escalating antigen dose (Fig. 10). The CS/D and NANP titers at 0.1 µg was significantly lower than all higher dose groups. However increase in titer from 1 to 10 µg was less pronounced, except that the NANP titer of 1 µg group was lower than the 10 µg group. Two weeks after the third immunization, mice were challenged with Tr-Pb sporozoites. All 7 mice in the PBS+GLA/SE adjuvant control group were positive by day six and only 1/7 mice in the 0.1 µg dose group was protected on day fourteen. As predicted by ELISA titers, higher protection was observed for the 1 µg (4/7 protected), 2.5 µg (6/7 protected), 5 µg (5/7) and 10 µg (6/7) dose groups (Fischer’s exact test p values comparing 0.1 µg against 1, 2.5, 5 and 10 µg groups were 0.2, 0.007, 0.03 and 0.007 respectively) (Table 2). The above data confirmed that GLA/SE-adjuvanted CS/D could induce potent antibody responses and protection against transgenic parasite challenge in mice.

### IgG2 associates with protection

**Figure 11:**
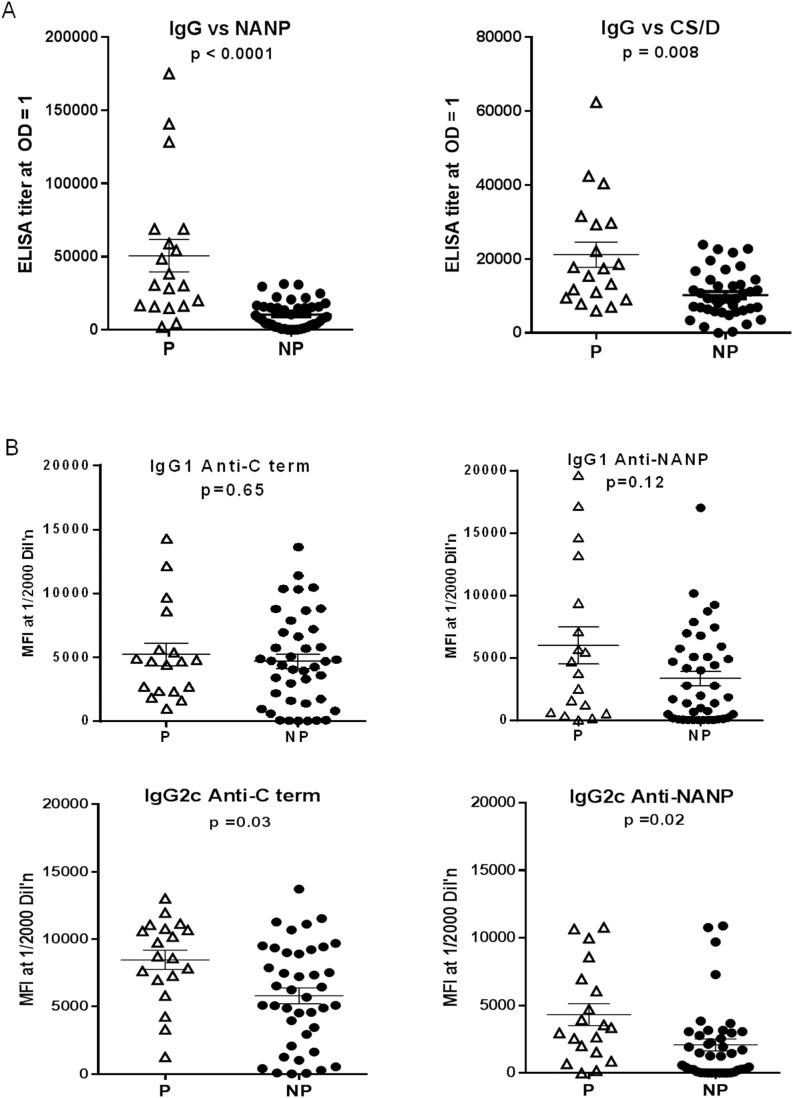
Comparison of serological profiles of protected and non-protected C57Bl/6 mice. Antibody data from the two C57Bl/6 mice challenge studies were combined and plotted separately for protected (P) mice and non-protected (NP) mice. *A,* NANP titers (left) or CS/D titers (right). *B,* IgG1 (upper panels) or IgG2c (bottom panels) subclass responses expressed as Luminex MFI, measured against the C term protein (left panels) or NANP repeat peptide (right panels). The p values are for two-way T-tests on log transformed data.

To study the role of antibodies in mediating protection, ELISA and subclass data of the challenged C57Bl/6 mice were plotted separately for protected and non-protected mice (Fig. 11). Protected mice had significantly higher total CS/D and NANP-specific IgG responses as compared to non-protected mice (Fig. 11A). When antibody subclasses were further dissected, protection was significantly associated with high levels of IgG2c against NANP and C term while IgG1 antibody levels failed to distinguish the protected and non-protected groups (Fig. 11B). No difference in the avidity of protected and non-protected mice was observed (not shown).

### TLR agonists increase CD86 production on B cells

Both T and B cell activation is controlled by a series of co-stimulatory molecules. Prominent among these is CD86, a central co-stimulatory molecule that augments the activation of T cells and is up-regulated by cytokines. As a preliminary exploration of a mechanism underlying GLA and R848 amplification of humoral and cell-mediated immunity, the effects of the TLR4 and TLR7/8 agonists on the expression of CD86 on B cells were investigated. *In vitro* exposure of murine spleen cells to the adjuvants GLA/SE or GLA/R848/SE produced a marked increase in expression of the co-stimulatory CD86 molecule on CD19^+^ B cells (Figure S3). A similar increase in CD86 was observed in the presence of the CS/D-3M051 while CS/D in the absence of the conjugated 3M051 had no measurable effect on CD86 levels (Figure S3).

## Discussion

The native *P. falciparum* CSP gene codes for an N-terminal segment which is followed by 4 NVDP repeats, 38 NANP repeats, and a C-terminal cysteine rich region (Fig. 2A). Although the central repeat and C-terminal regions contain the primary B and T cell epitopes [39], several T helper and CTL epitopes have been mapped to the N-terminal region [32], [40], [41]. The N-terminal also plays a key role in the process of sporozoite invasion and inclusion of the N-terminal region could be a way to improve the efficacy of CSP based vaccines [42] [38]. High level expression of a nearly full-length CSP in *E. coli* has however remained a major hurdle. Of the 3 reports on full-length *P. falciparum* CSP expression, the first recounted that the full-length CSP gene cannot be expressed in *E. coli* [43] and the two subsequent reports showed expression of an insoluble protein that required extensive refolding to gain solubility [36], [44]. We demonstrate that a nearly full-length PfCSP construct can be expressed and purified from the soluble fraction of *E. coli*. The final GMP product met the purity, homogeneity and stability criteria for clinical use products and it reacted to both conformational and non-conformational CSP specific monoclonal antibodies (Fig. 4 and Table 1). A comparative immunogenicity study was next conducted to find a suitable adjuvant for the soluble cGMP grade CS/D antigen.

Given the safety and regulatory concerns for vaccines primarily targeted for use in children, any combination of novel adjuvants would need to demonstrate significant enhancement of CSP-specific immunity for serious consideration towards advanced development. We tested TLR4 agonist containing adjuvants GLA/SE and two TLR7/8 agonists containing adjuvants R848 (soluble) and 3M051 (conjugated). The 3M051 adjuvant offered no improvement over GLA/SE in the C57Bl/6 protection model. Additionally, the 3M051 formulation would require another GMP conjugation, increasing manufacturing costs for a vaccine targeted at low resource countries. Hence, our study data favors the use of GLA/SE over 3M051/SE as an adjuvant for soluble CS/D. Contrary to the TLR synergy hypothesis, adding R848 and 3M051 to GLA/SE showed no improvement in antibody responses or subclass profile, relative to 3M051/SE or GLA/SE administered individually. This lack of synergy could be because the dose of the two adjuvants was relatively high. Moreover, TLR4 signals through both the TRIF and MyD88 pathways [12], [45] and synergistic combinations of GLA with the TRIF pathway ligand Poly(I-C) may be worth exploring with CSP vaccines [46].

Kastenmuller *et al.* compared poly-ICLC (a TLR3 agonist) to GLA/SE for its capacity to induce an immune response to nearly full-length CS proteins in the rodent model. In that study, poly-ICLC was found to induce higher T-cell responses than GLA/SE, although no differences in antibody responses were reported [46]. If antibodies are a prime mediator of CSP-based protection, as was determined in past RTS,S vaccine studies [5], GLA/SE is likely to be at least as effective as poly-ICLC. We showed that the GLA/SE formulations of CS/D induced a robust antibody response, measureable T-cell responses and 86% protection in mice that received three doses of 2.5 µg *E. coli*-produced CS/D (Table 2). While only a head-to-head comparison of vaccine formulations can be definitive, it is notable that three doses of the poly-ICLC formulation containing 20 µg of a similar *E. coli* produced CSP showed about 50% sterile protection against a CSP repeat transgenic *P. berghei* parasite [46].

In our attempts to study the capacity of adjuvants to promote immune responses to our CS/D vaccine, it became evident that magnitude of total IgG as measured by ELISA was not sufficient to reveal the essential features of the antibody response. Although our experimental design did not allow us to dissect the role of cellular immunity, we show for the first time that CS/D-specific IgG2c levels were significantly higher in protected C57Bl/6 mice as compared to non-protected mice while IgG1 titers did not appear to associate with protection. Although the CS/D-vaccinated Balb/c mice were not challenged, GLA/SE and GLA/R848/SE also stimulated much stronger IgG2a, but weaker IgG1 responses than SE in this mouse strain.

A trend towards increased protection from re-infection with malaria has been noted in individuals who possess higher levels of cytophilic, CSP-specific antibodies in the field [47]. In mice, the IgG2 subclass is cytophilic, as it can fix complement [48] and opsonize pathogens for phagocytosis more effectively than IgG1 [49]. In humans, it is the IgG1 and IgG3 subclasses that are cytophilic, while IgG2 and IgG4 have been shown to inhibit cytophilic activity [50]. This study did not find a negative correlation between IgG1 responses and protection in the mouse model and unlike 3M051, GLA/SE was found to promote high levels of both IgG2 and IgG1, in two strains of mice. The overall results suggest that that our *E. coli*-derived CS/D adjuvanted with GLA/SE merits further evaluation in the rhesus monkey model and subsequently in humans.

## Supporting Information

Figure S1 **Representative titration curve for Luminex dilution determination.** Figure shows titration of representative high and low responder mice in the GLA/SE and SE groups from the 3 vaccination C57Bl/6 challenge experiment. The dotted black line was drawn at the 1∶2000 dilution, selected for further analysis on all sera.
(TIF)

Figure S2 **Gating scheme for intra-cellular staining of T cells from Balb/c mice.** Mice were vaccinated with 2 doses of a CS/D-3M051 formulation and 6 weeks after the final vaccination splenic lymphocytes were extracted and stimulated with CS/D or the CS protein K^d^-restricted peptide (CD8). *A,* Total cell population (> = 300,000 cells/sample) was gated for viable cells, CD3 T cells and CD4 or CD8 T cells. *B,* Representative dot plots showing the expression of IFN-γ in CD44^+^CD4^+^ naïve cells cultured with CS/D, immune cells cultured in culture medium only (CM) or immune cells cultured with CS/D**.** *C,* Representative dot plots showing the expression of IFN-γ in CD8^+^ naïve cells cultured with CS/D, immune cells cultured in culture medium only or.
(TIF)

Figure S3 **Adjuvant-mediated up-regulation of CD86 in C57Bl/6 mice.** Left, CD86 expression on CD19^+^ B cells from naïve C57Bl/6 mice were incubated overnight (see methods) with medium alone (red), 1 µg/ml GLA/SE (blue) or 1 µg/ml of GLA/R848/SE (green). Right, medium alone (red), 1 µg/ml CS/D (blue) or 1 µg/ml CS/D-3M051 conjugate (green).
(TIF)
