# Supplementary figures and images for: IgG2 Antibodies against a Clinical Grade Plasmodium falciparum CSP Vaccine Antigen Associate with Protection against Transgenic Sporozoite Challenge in Mice
Source: PLoS One. 2014 Oct 24;9(10):e111020. doi: 10.1371/journal.pone.0111020 (PMC4208815; doi:10.1371/journal.pone.0111020)

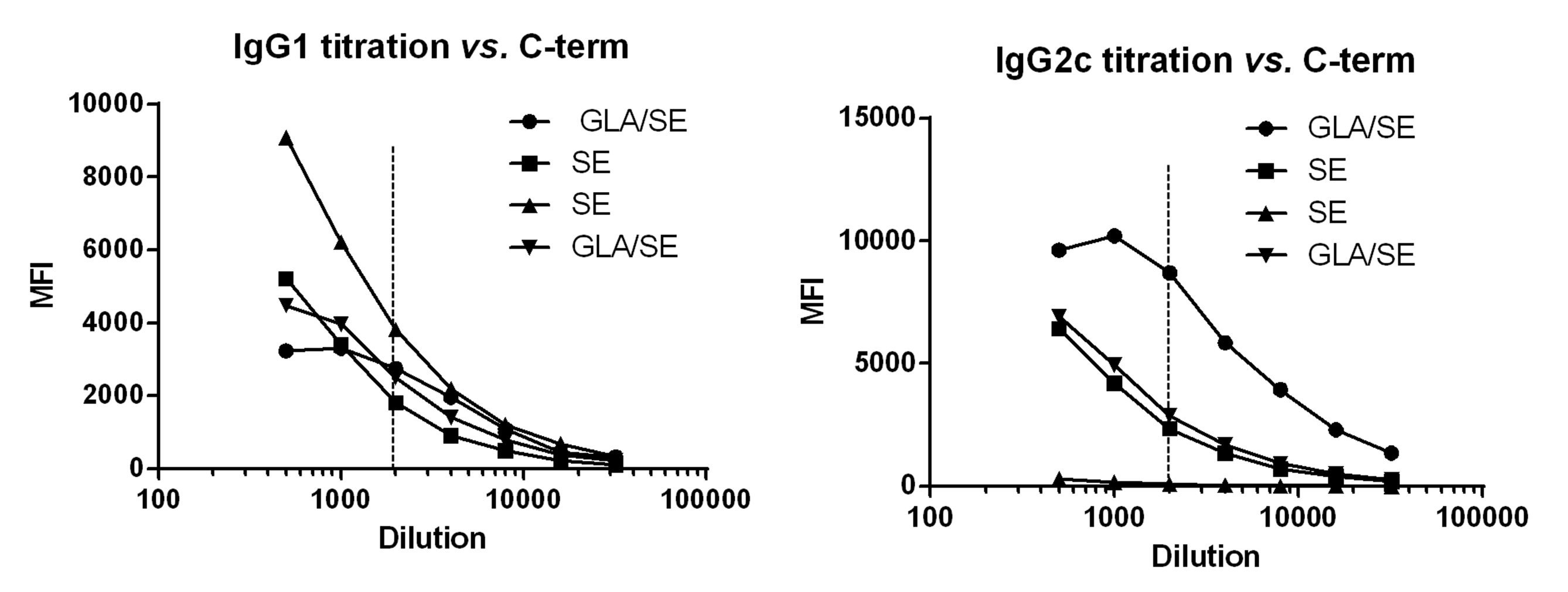

Supplement: Figure S1 — Representative titration curve for Luminex dilution determination. Figure shows titration of representative high and low responder mice in the GLA/SE and SE groups from the 3 vaccination C57Bl/6 challenge experiment. The dotted black line was drawn at the 1∶2000 dilution, selected for further analysis on all sera. (TIF) [file pone.0111020.s001.tif]

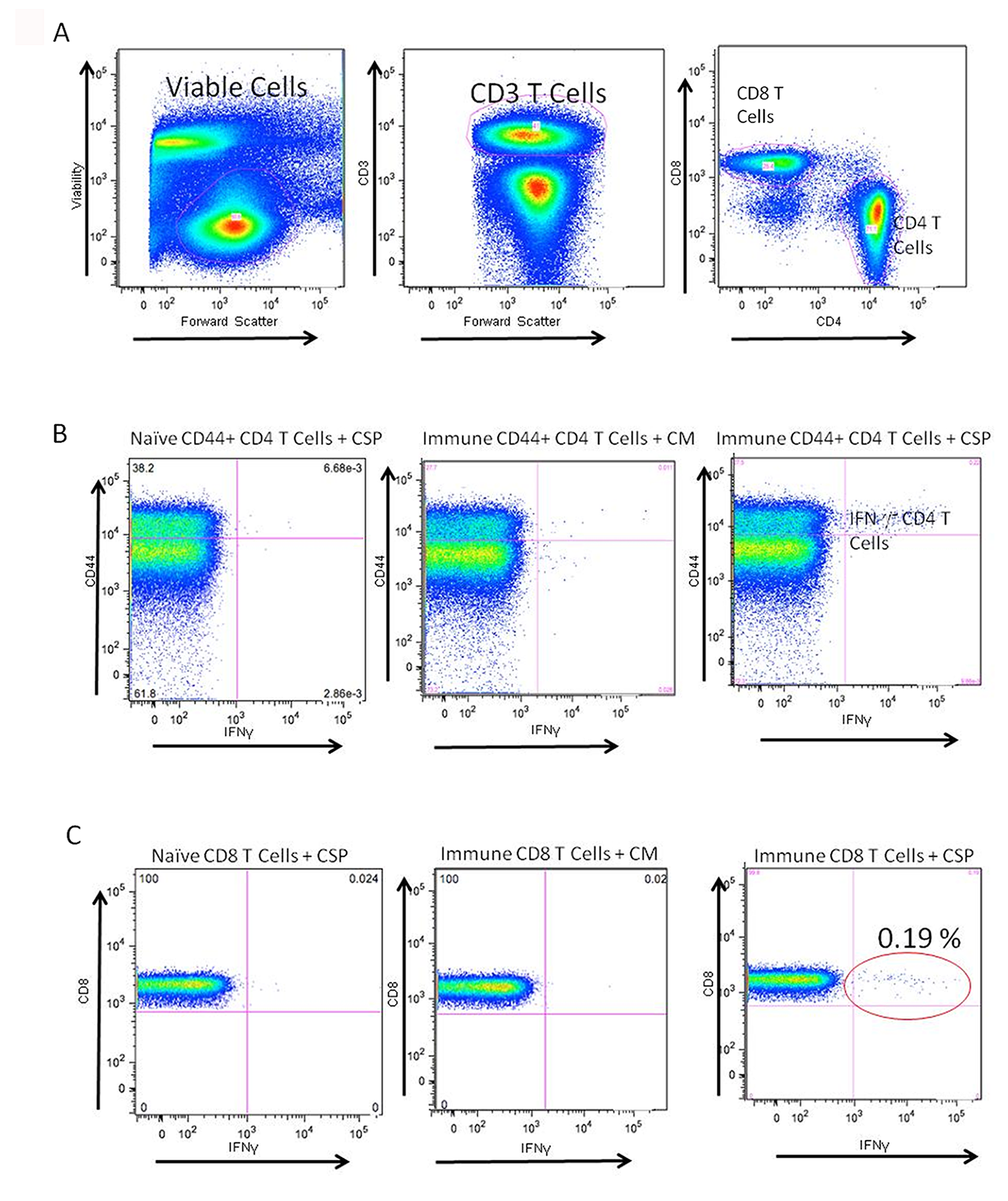

Supplement: Figure S2 — Gating scheme for intra-cellular staining of T cells from Balb/c mice. Mice were vaccinated with 2 doses of a CS/D-3M051 formulation and 6 weeks after the final vaccination splenic lymphocytes were extracted and stimulated with CS/D or the CS protein Kd-restricted peptide (CD8). A, Total cell population (> = 300,000 cells/sample) was gated for viable cells, CD3 T cells and CD4 or CD8 T cells. B, Representative dot plots showing the expression of IFN-γ in CD44+CD4+ naïve cells cultured with CS/D, immune cells cultured in culture medium only (CM) or immune cells cultured with CS/D. C, Representative dot plots showing the expression of IFN-γ in CD8+ naïve cells cultured with CS/D, immune cells cultured in culture medium only or. (TIF) [file pone.0111020.s002.tif]

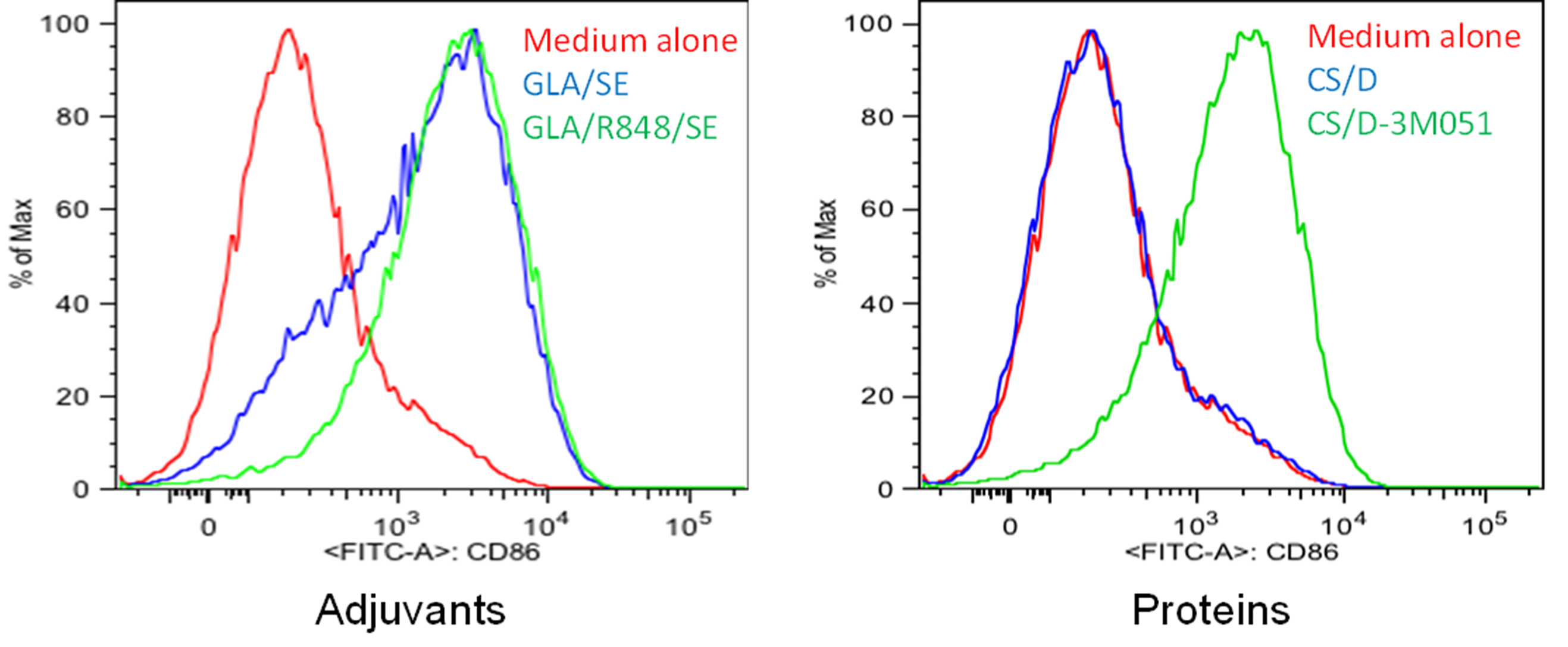

Supplement: Figure S3 — Adjuvant-mediated up-regulation of CD86 in C57Bl/6 mice. Left, CD86 expression on CD19+ B cells from naïve C57Bl/6 mice were incubated overnight (see methods) with medium alone (red), 1 µg/ml GLA/SE (blue) or 1 µg/ml of GLA/R848/SE (green). Right, medium alone (red), 1 µg/ml CS/D (blue) or 1 µg/ml CS/D-3M051 conjugate (green). (TIF) [file pone.0111020.s003.tif]
